# Cultural and demographic correlates of dual tobacco use in a sample of Alaska Native adults who smoke cigarettes

**DOI:** 10.18332/tid/122902

**Published:** 2020-06-16

**Authors:** Anna E. Epperson, Maria Crouch, Jordan Skan, Neal L. Benowitz, Matthew Schnellbaecher, Judith J. Prochaska

**Affiliations:** 1 Division of Adolescent Medicine, Department of Pediatrics, Stanford University School of Medicine, Stanford, United States; 2 Cardiology Department, Alaska Native Tribal Health Consortium, Anchorage, United States; 3 Program in Clinical Pharmacology, Division of Cardiology, Department of Medicine, University of California San Francisco, San Francisco, United States; 4 Stanford Prevention Research Center, Department of Medicine, Stanford University, Stanford, United States

**Keywords:** cigarettes, tobacco, dual/poly-tobacco use, Alaska Native

## Abstract

**INTRODUCTION:**

Approximately 9 million American adults use two or more tobacco products regularly, referred to as dual or poly tobacco users. In Alaska, where tobacco is not native, approximately 20% of the population smokes cigarettes, and among smokers, 10% use two or more tobacco products. Previous research suggests that dual tobacco product use may be especially high among Alaska Native people. The current study examined cultural and demographic characteristics associated with dual tobacco use.

**METHODS:**

Alaska Native adults reporting daily smoking and identified with high blood pressure or cholesterol were recruited in the Norton Sound region of Alaska between 2015–2019 as part of a treatment trial targeting cardiovascular disease risk factors. Participants reported their tribal group, level of identification with their Alaska Native heritage, speaking of their tribal language, basic demographic characteristics, and past 30-day use of tobacco products in addition to smoking cigarettes.

**RESULTS:**

Participants (n=299) were 48.5% female and identified as Yup’ik (31.1%), Inupiat (60.5%), and other or multiple tribal group(s) (8.4%). Most participants (85.3%) strongly identified with their Alaska Native heritage. Past 30-day dual tobacco use was reported by 10.0%, specifically 9.0% chew/snuff, 1.3% e-cigarettes, and 0.7% Iq’mik. Multivariate regression models indicated that dual tobacco use was more likely among men (OR=3.35; 95% CI: 1.30–8.64), younger participants (OR range: 10.97–12.35; 95% CI: 2.33–57.86), those identifying as Yup’ik (OR=2.86; 95% CI: 1.13–7.19), and those who identified very little or not at all with their Alaska Native heritage (OR=2.98; 95% CI: 1.14–7.77).

**CONCLUSIONS:**

Young men identifying as Yup’ik were more likely to use dual forms of tobacco. Stronger identification with one’s Alaska Native heritage was associated with lower risk of dual tobacco use. The findings highlight cultural and demographic factors for further consideration and attention in tobacco cessation treatment interventions.

## INTRODUCTION

Regular use of two or more tobacco products (e.g. cigarettes, cigars, smokeless tobacco, e-cigarettes), termed dual or poly use^1^, is common^2^. Approximately 9 million adults in the US use two tobacco products or more^2^, with the most prevalent dual use combinations of cigarettes/e-cigarettes (30.1%) and cigarettes/cigars (29.2%)^3^. Recent data indicate that dual tobacco use may be on the rise^3,4^, may be associated with lower quit rates^5^, and may result in greater exposure to tobacco toxins^1^.

Although smoking in the US has significantly declined over the last several decades, disparities exist. Compared to all other major racial/ethnic groups, American Indian/Alaska Native people have the highest prevalence of any tobacco use (32.3% vs 21.9% or less)^2^, and specifically cigarette smoking (22.6% vs 15.0% or less)^2^ and smokeless tobacco use (8.4% vs 4.5% or less)^6^. Among American Indian/Alaska Native people, 4.9% report dual tobacco product use compared to 4.1% or less for other major racial/ethnic groups^6^.

Specifically, in Alaska, 35.1% of Alaska Native people smoke cigarettes compared to 20.7% or less for other major racial/ethnic groups^7^. The higher smoking prevalence is due in part to low quit rates among Alaska Native adults who smoke^8^. Regional differences in tobacco product use also are apparent. State surveillance data indicate that cigarette smoking among Alaska Native adults is higher in the Northern part of Alaska (39.1%) compared to other regions (≤34.4%)^7^. Notably, 49.3% of Alaska Native adults in the Norton Sound region of Alaska smoke cigarettes compared to 39.0% among Alaska Native adults in Alaska’s southwest Yukon-Kuskokwim region^7^; whereas smokeless tobacco use prevalence in Norton Sound is lower (10.4%) than that in the Yukon-Kuskokwim region (37.5%)^9^. Among all adults in Alaska who smoke, 10% report dual tobacco product use)^8^, while estimates of dual use among Alaska Native people who smoke range from 3.0% to 11.0%, differing by region and demographic characteristics^10-12^.

Tobacco use is a major risk factor for the leading causes of preventable death among Alaska Native people: cardiovascular disease and cancer^13,14^. Tobacco is not native to Alaska, but rather was brought to the region through colonization with trade^15^. Alaska Native people comprise roughly 15.4% of Alaska’s state population^16^ with most Alaska Native people still living near or on their original homelands. With distinct tribal cultures by region (e.g. Inupiat in Northeast and Arctic, Tlingit and Haida in Southeast Alaska), there are approximately 224 federally-recognized tribes in Alaska and at least 20 tribal languages spoken^17^.

Despite the co-occurring use of tobacco products and that all tobacco products are harmful to health^13^, most tobacco treatment (cessation) programs are focused on reducing and eliminating cigarette smoking without attention to other forms of tobacco. To inform treatment approaches, we sought to better understand factors associated with dual tobacco product use, with a focus on treatment development for Alaska Native communities. Given epidemiologic data indicating particularly high prevalence of tobacco use in the Northern part of Alaska, we studied dual tobacco use among Alaska Native adults within the Norton Sound region and examined demographic and cultural correlates as well as associations with smoking behaviors.

## METHODS

### Participants and procedure

Participants were adults reporting daily smoking and identified as having high blood pressure or high cholesterol. The sample was recruited as part of a randomized clinical trial targeting cardiovascular disease risk behaviors in Alaska Native people in the Norton Sound region^18^. Participants were randomized to one of two active-intervention arms promoting: 1) tobacco cessation and increased physical activity; or 2) medication adherence and a heart-healthy Alaska Native diet. All participants received motivational, stage-tailored, telemedicine-delivered counseling sessions four times in a 12-month period. The Norton Sound region is located in Northwestern Alaska with about 75.0% of the population of Alaska Native heritage, primarily identifying as Inupiat and Yup’ik^19^.

Participants were recruited through intensive community outreach (e.g. public radio announcements, flyers, study information letters mailed to patients from the local clinic providers, tabling in high-traffic community areas). Eligibility criteria included high blood pressure (systolic/diastolic BP ≥140 mmHg/90 mmHg) or high cholesterol (LDL ≥160 mg/dL), residence in the Norton Sound region, aged ≥19 years, and smoking at least five cigarettes daily. Exclusion criteria were current participation in a tobacco treatment program, taking cessation medications, a body mass index of ≥50 (kg/m^2^), and active pregnancy. All participants provided informed consent. Study procedures were approved by the Institutional Review Boards at Stanford University; the University of California, San Francisco; the Alaska Area Institutional Review Board; the Alaska Native Tribal Health Consortium Board and its manuscript and proposal review committee; and the Norton Sound Health Corporation Board of Directors and its Research Ethics Review Board. Baseline data analyzed here were collected June 2015 through January 2019.

### Measures

#### Demographic characteristics

Participants reported their age, gender, level of education, annual household income, and subjective social status. Age was categorized as 19–35, 36–50, ≥51 years old. Education level attained was analyzed as some high school or less, high school graduate, or some college or more. Annual household income was categorized as ≤10000, 11000–25000, 26000–50000 and ≥51000 US$. Subjective social status in one’s community was reported on a scale from 1 (lowest) to 10 (highest)^20^.

#### Alaska Native cultural characteristics

Participants reported their Alaska Native tribal group (Aleut, Athabascan, Inupiat, Yup’ik, other) and whether they spoke their tribal language (no/yes). Due to low numbers of Aleut and Athabascan participants, analyses compared Inupiat only, Yup’ik only, and other. Participants who identified with more than one tribal group (n=18) were classified as other. Participants also reported their level of identification with their Alaska Native heritage with response options dichotomized for analyses as not at all/very little and somewhat/very much.

#### Smoking characteristics

Smoking characteristics included the average number of cigarettes smoked per day in the past week, smoking of menthol cigarettes (analyzed as non-menthol only vs menthol-only or both), the age of smoking initiation, time to first cigarette upon waking (analyzed as within or after 30 minutes), and home smoking rules (analyzed as no smoking allowed indoors or other). Lifetime 24-h quit smoking attempts were analyzed as none or one or more. Stage of change for quitting smoking was categorized as in precontemplation (not intending to quit in the next 6 months), contemplation (intending to quit in the next 6 months), or preparation stage of change (prepared to quit in the next month and made a 24-h quit attempt within the past year)^21^.

#### Dual tobacco use

All participants were recruited as smoking cigarettes daily. In addition, we assessed ever use on an everyday or almost everyday basis of commercial chew/snuff, cigars, Iq’mik (i.e. ash from the birch tree fungus mixed with tobacco leaves to be chewed)^22^, and e-cigarettes. Those who reported daily or almost daily ever use of another tobacco product were asked when they last used the product. In the presence of daily smoking, dual tobacco use in our sample was defined as use of at least one of the products (chew/snuff, cigars, Iq’mik, or e-cigarettes) every day/almost every day within the past month.

### Data Analysis

Demographic, cultural, and smoking characteristics were examined for differences by dual tobacco product use status using independent samples t-tests for continuous variables and chi-squared tests for categorical variables. A family-wise Bonferroni correction was applied for three conceptual variable groups: 1) demographic (5 items, p<0.010); 2) cultural (3 items, p<0.017); and 3) smoking (6 items, p<0.008) characteristics. Significant univariate correlates were analyzed together in a multivariate logistic regression model. Missing data for the univariate analyses were minimal (<5%). Only one participant was excluded from model testing in the final regression model due to missing data.

## RESULTS

Table 1 summarizes sample descriptive characteristics overall and by dual tobacco product use. In the sample of 299 participants, 27.8% were aged 19–35 years, 29.1% 36–50 years, and 43.1% ≥51 years; 51.5% identified as male. Alaska Native tribal group identification was Inupiat only (60.5%), Yup’ik only (31.1%), and other (8.4%); 61.1% of participants spoke their Alaska Native tribal language. Most participants (85.3%) somewhat or very much identified with their Alaska Native heritage.

**Table 1:** Demographic, cultural and smoking characteristics of the sample by dual tobacco use status, Alaska, USA, 2015–2019 (N=299)

*Characteristics*	*Overall (n=299)*	*No dual use (n=269)*	*Dual use (n=30)*
**All participants,** %	100	90.0	10.0
**DEMOGRAPHIC**			
**Age** (years), %			
19–35	27.8	25.7^a^	46.7^b^
36–50	29.1	27.1^a^	46.7^b^
≥51	43.1	47.2^a^	6.7^b^
**Gender**, %			
Female	48.5	51.3^a^	23.3^b^
**Education level**, %			
Some high school or less	20.5	19.4	30.0
High school graduate	59.7	60.4	53.3
Some college or more	19.8	20.1	16.7
**Annual household income** (US$), %			
≤10000	29.4	29.2	31.0
11000–25000	21.3	21.3	20.7
26000–50000	20.6	21.0	17.2
≥51000	9.5	10.5	0
Don’t know/refused	19.3	18.0	31.0
**Community subjective social status**, mean (SD)	5.3 (2.1)	5.3 (2.1)	5.2 (1.8)
**CULTURAL**			
**Alaska Native tribal group,** %			
Inupiat	60.5	63.2^a^	36.7^b^
Yup’ik	31.1	29.4	46.7
Other	8.4	7.4	16.7
**Identification with Alaska Native heritage**, %			
Not at all/very little	14.4	12.3^a^	33.3^b^
Very much/somewhat	85.3	87.7^a^	66.7^b^
**Speaks Alaska Native tribal language**, %	61.1	60.4	66.7
**SMOKING**			
**Cigarettes smoked per day**, mean (SD)	12.4 (10.0)	12.6 (10.4)	11.3 (6.5)
**Smokes ≥10 cigarettes per day**, %	63.5	64.2	60.0
**Age at smoking initiation,** mean (SD)	16.8 (5.6)	17.0 (5.7)	15.1 (4.3)
**Smokes within 30 minutes of waking,** %	65.3	64.2	75.9
**Home indoor smoking ban**, %	13.8	12.6	23.3
**Reported 24-h lifetime quit attempt,** %	80.3	84.0	83.3
**Stage of change for smoking cessation,** %			
Precontemplation	32.8	33.8	30.0
Contemplation	45.8	46.4	50.0
Preparation	19.4	19.8	20.0

a,b Denote significant group differences for the characteristic of interest (rows) by dual tobacco (columns) per chi-squared and t-test analyses.

A family-wise Bonferroni correction was applied for three conceptual variable groups: 1) demographic (5 items, p<0.010); 2) Alaska Native cultural (3 items, p<0.017); and 3) smoking behaviors (6 items, p<0.008) characteristics. SD: standard deviation.

Among the 299 participants, 30 (10.0%) reported past month dual use, with 9.0% using chew tobacco/snuff, 1.7% Iq’mik, and 0.3% e-cigarettes; no one reported current regular use of cigars. In univariate analyses, significant correlates of dual tobacco product use were being male, of younger age, identifying as Yup’ik, and identifying very little or not at all with one’s Alaska Native heritage (Table 1). The other demographic and all smoking behavior variables were not significantly associated with dual tobacco use.

Analyzing the significant demographic and cultural correlates in a multivariate model, we found all remained statistically significant in association with dual tobacco product use (Table 2). In the multivariate logistic regression model, being male (OR=3.35; 95% CI: 1.30–8.64), aged 36–50 years (OR=12.35; 95% CI: 2.64–57.86) or 19–35 years (OR=10.97; 95% CI: 2.33–51.59) compared to older age (≥51 years), identifying as Yup’ik (OR=2.86; 95% CI: 1.13–7.19) relative to Inupiat, and weaker identification with one’s Alaska Native heritage (OR=2.98; 95% CI: 1.14–7.77) were significantly associated with a greater likelihood of dual tobacco product use.

**Table 2:** Multivariate logistic regression model for dual use of another tobacco product, Alaska, USA, 2015–2019 (N=298)^a^

*Characteristics*	*n*	*OR (95% CI)*
**Age** (years)		
≥51 (Ref.)	129	1.00
36–50	86	12.35 (2.64–57.86)*
19–35	83	10.97 (2.33–51.59)*
**Gender**		
Female (Ref.)	145	1.00
Male	153	3.35 (1.30–8.64)*
**Alaska Native tribal group**		
Inupiat (Ref.)	181	1.00
Yup’ik	93	2.86 (1.13–7.19)*
Other	24	3.36 (0.94–12.04)
**Identification with Alaska Native heritage**		
Very much/somewhat (Ref.)	255	1.00
Not at all/very little	43	2.98 (1.14–7.77)*

OR: odds ratio. CI: confidence interval.

* Statistically significant at p<0.05, controlling for covariates in the table.

a One participant was excluded from model testing in the final regression model due to missing data.

## DISCUSSION

The current study examined demographic and cultural characteristics associated with past month dual tobacco use among Alaska Native adults reporting daily smoking. One in ten participants reported use of another tobacco product in addition to cigarettes, which matches the prevalence of dual use among adults who smoke cigarettes in the state overall^8^. At the national level, the most common pairs of tobacco products dually used were cigarettes and e-cigarettes followed by cigarettes and cigars^3^. In contrast, none of the participants in the current study reported current regular use of cigars, and current e-cigarette use was very low (<1%). Instead, the most common other tobacco product use in our sample was commercial smokeless tobacco (e.g. chew, snuff). Iq’mik, another form of smokeless tobacco, was reported by few in our sample.

Dual tobacco use in our sample was more common among younger than older adults and among males than females. While prior research has identified younger age and gender in association with dual tobacco product use^11,12^, a study of Western Alaska Native people found greater dual use among females than males, with particular high prevalence of Iq’mik use among the women in their sample^12^. Differences in findings by gender may be due to regional differences in tobacco product use. As noted, commercial smokeless tobacco was the most common other tobacco product used by our sample, and Alaska state data indicate that smokeless tobacco use generally is more common among Alaska Native men compared to women^23^.

Controlling for gender and age, dual tobacco use was found to vary by Alaska Native cultural factors including tribal identification. Participants who identified as Yup’ik, rather than Inupiat, were more likely to use another tobacco product in addition to smoking cigarettes. The burden of dual tobacco use may be specific to certain tribal groups or communities rather than Alaska Native people generally. Notably, participants who more strongly identified with their Alaska Native heritage were less likely to use another tobacco product, indicating a potential protective factor. We believe these findings are novel and worthy of further investigation.

In the current sample, dual tobacco use was not significantly associated with participants’ smoking characteristics including the age of smoking initiation, the number of cigarettes smoked per day, time to first cigarette upon waking, intention to quit and past quit attempts, or home smoking rules. In contrast, a prior study of dual tobacco use in southwestern Alaska found that dual use was associated with smoking fewer cigarettes per day and, as a result, lower cigarette dependence scores^11^.

Tobacco use remains the leading preventable cause of morbidity and mortality in the US^13^ and is especially prevalent among Alaska Native people where over a third currently smoke cigarettes^7^. The current findings may have implications for tobacco control policy and treatment efforts. Findings highlight the relevance of cultural factors to dual tobacco use. Given that tobacco use is not native to the region, efforts that build connection to and identification with one’s Alaska Native culture may bring the added benefit of dissuading tobacco use and supporting tobacco cessation. Given the differences observed here by tribe in the prevalence of dual tobacco product use, attention to regional and tribal differences is warranted.

### Strengths and limitations

Study strengths include the inclusion of multiple cultural factors and a detailed assessment of tobacco-related product use and related behaviors. Data were collected in only one Northern Alaska region. Findings may differ for Alaska Native people in other regions where the use of other types of tobacco (e.g. Iq’mik) may be more prevalent. Participants were all adult daily smokers with at least one cardiovascular disease risk factor; hence, findings may have limited generalizability. Data for the current study were cross sectional, and therefore, it is not possible to draw any casual connections. Future research should examine changes over time in dual product use in the context of demographic, Alaska Native cultural, and tobacco-related behaviors.

## CONCLUSIONS

We found a higher likelihood of dual tobacco use among young adults, males, and those identifying as Yup’ik. In contrast, strongly identifying with one’s Alaska Native heritage was associated with a lower likelihood of dual tobacco use. More research is needed to clarify the underlying mechanisms of the relationship between Alaska Native cultural factors and dual tobacco product use. Strategies that seek to strengthen tribal group identity should be included as part of current tobacco prevention and treatment and may be a promising method to help decrease tobacco use.
